# Quantifying Distribution of Flow Cytometric TCR-Vβ Usage with Economic Statistics

**DOI:** 10.1371/journal.pone.0125373

**Published:** 2015-04-29

**Authors:** Kornelis S. M. van der Geest, Wayel H. Abdulahad, Gerda Horst, Pedro G. Lorencetti, Johan Bijzet, Suzanne Arends, Marieke van der Heiden, Anne-Marie Buisman, Bart-Jan Kroesen, Elisabeth Brouwer, Annemieke M. H. Boots

**Affiliations:** 1 Department of Rheumatology and Clinical Immunology, University of Groningen, University Medical Center Groningen, Groningen, The Netherlands; 2 Center for Immunology of Infectious Diseases and Vaccines, National Institute for Public Health and the Environment, Bilthoven, The Netherlands; 3 Department Laboratory Medicine, University of Groningen, University Medical Center Groningen, Groningen, The Netherlands; University of Melbourne, AUSTRALIA

## Abstract

Measuring changes of the T cell receptor (TCR) repertoire is important to many fields of medicine. Flow cytometry is a popular technique to study the TCR repertoire, as it quickly provides insight into the TCR-Vβ usage among well-defined populations of T cells. However, the interpretation of the flow cytometric data remains difficult, and subtle TCR repertoire changes may go undetected. Here, we introduce a novel means for analyzing the flow cytometric data on TCR-Vβ usage. By applying economic statistics, we calculated the Gini-TCR skewing index from the flow cytometric TCR-Vβ analysis. The Gini-TCR skewing index, which is a direct measure of TCR-Vβ distribution among T cells, allowed us to track subtle changes of the TCR repertoire among distinct populations of T cells. Application of the Gini-TCR skewing index to the flow cytometric TCR-Vβ analysis will greatly help to gain better understanding of the TCR repertoire in health and disease.

## Introduction

A broad T cell receptor (TCR) repertoire is considered critical for optimal immunity against a wide variety of antigens [1,2]. Contraction of the TCR repertoire is associated with poor vaccine responses in aged individuals [3], progression to AIDS in HIV-infected patients [4], and poor survival in cancer patients [5]. Assessment of the T cell receptor (TCR) repertoire is therefore relevant to researchers in many fields of medicine.

Several techniques are currently used to study the TCR repertoire [6]. Moderate to high resolution assessment of the TCR repertoire is provided by PCR-based methods, such as TCR spectratyping and sequencing. As these methods are relatively labor-intensive and preferably require cell-sorting of highly pure T cell populations, many researchers turn to flow cytometry [6]. Flow cytometry quickly measures the proportional TCR-Vβ usage in multiple T cell subsets on a per-cell basis, without the need for cell-sorting. Although this assessment can provide helpful information, an accurate and reliable way for analyzing the flow cytometric data on the TCR repertoire is currently lacking.

**Fig 1:**
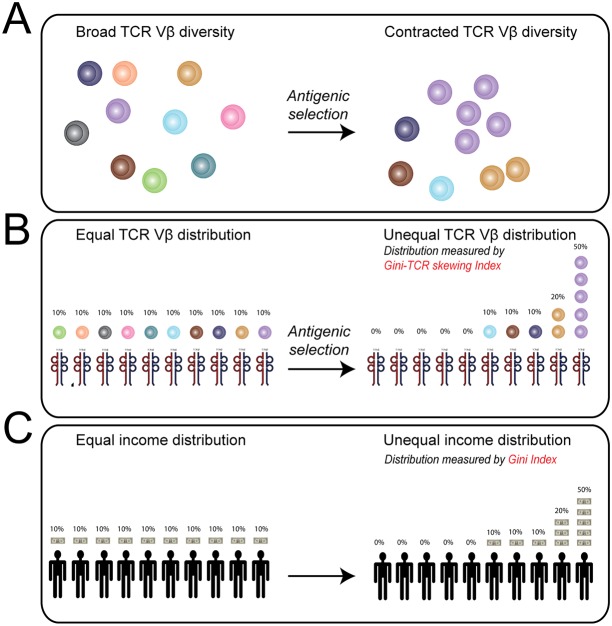
Schematic overview showing the relation between T cell receptor (TCR) Vβ diversity, distribution and percentages. (A) Schematic drawing illustrating broad versus contracted TCR-Vβ diversity. (B) Schematic drawing illustrating distribution and proportional usage of TCR Vβ families when TCR-Vβ diversity is broad or contracted. (C) Schematic drawing illustrating distribution of income among people.

Here, we introduce economic statistics to improve the analysis of flow cytometric data on TCR-Vβ usage. We noticed that the distribution of TCR-Vβ families among T cells resembles the distribution of income among people (Fig 1A–1C). Economists typically study the distribution of income by constructing Lorenz curves and calculating the Gini index. The Gini index, with scores ranging from 0 to 100, is a direct measure of income distribution [7–9]. By applying the Gini index to the flow cytometric TCR-Vβ analysis, we could directly measure the distribution of 24 TCR-Vβ families among multiple, well-defined T cell subsets. In this context, low Gini index values indicated equal distribution of TCR-Vβ families (i.e. broad repertoire), whereas high values pointed to unequal distribution of TCR-Vβ families (i.e. repertoire skewing). Although the Gini index has been used in TCR sequencing studies [10,11], we here demonstrate that the Gini index, hence referred to as the Gini-TCR skewing index, also aids the analysis of flow cytometric data on TCR-Vβ usage. Importantly, the Gini-TCR skewing index allowed us to detect subtle changes of the TCR repertoire among multiple, well-defined T cell subpopulations.

## Methods

### Subjects

Heparinized blood samples were acquired from 27 healthy volunteers. Eight men and 19 women were included (age range 22–81). Samples of 5 children (age 9 years) undergoing DTaP-IPV vaccination were also collected. Written informed consent was obtained from adult volunteers or from parents on behalf of the children. The study was approved by the institutional review board of the UMCG (METc2012/375) and the Central Committee on Research Involving Human Subjects in the Netherlands (CCMO; ISRCTN64117538). None of the study participants had an overt history of infection, cancer or auto-immune disease.

### Flow cytometry

Whole blood samples (150 μL) or isolated peripheral blood mononuclear cells were incubated with fluorochrome-conjugated antibodies (Table 1 and S1 Fig) for 45 minutes at room temperature. Subsequently, whole blood samples were lysed with 2 mL of 1x Lysing solution (BD Biosciences) for 10 minutes at room temperature. Finally, samples were washed twice with phosphate buffered saline containing 1% bovine serum albumin. Samples were measured immediately on a LSR-II flow cytometer (BD Biosciences). The flow cytometric data was obtained with FACS Diva (BD Biosciences) and analyzed with Kaluza Software (Beckman Coulter). Fluorescence-minus-one (FMO) controls served as negative controls for the flow cytometric staining.

**Table 1:** Monoclonal antibodies.

Antibody specificity	Fluorochrome	Clone	Manufacturer
Vβ1	PE+FITC	BL37.2	Beckman Coulter
Vβ2	PE+FITC	MPB2D5	Beckman Coulter
Vβ3	FITC	CH92	Beckman Coulter
Vβ4	PE+FITC	WJF24	Beckman Coulter
Vβ5.1	PE+FITC	IMMU157	Beckman Coulter
Vβ5.2	PE	36213	Beckman Coulter
Vβ5.3	PE	3D11	Beckman Coulter
Vβ7.1	PE+FITC	ZOE	Beckman Coulter
Vβ7.2	FITC	ZIZOU4	Beckman Coulter
Vβ8	FITC	56C5.2	Beckman Coulter
Vβ9	PE	FIN9	Beckman Coulter
Vβ11	PE	C21	Beckman Coulter
Vβ12	FITC	VER2.32	Beckman Coulter
Vβ13.1	PE	IMMU222	Beckman Coulter
Vβ13.2	PE	H132	Beckman Coulter
Vβ13.6	PE+FITC	JU74.3	Beckman Coulter
Vβ14	FITC	CAS1.1.3	Beckman Coulter
Vβ16	FITC	TAMAYA1.2	Beckman Coulter
Vβ17	PE+FITC	E17.5F3	Beckman Coulter
Vβ18	PE	BA62.6	Beckman Coulter
Vβ20	FITC	ELL1.4	Beckman Coulter
Vβ21.3	FITC	IG125	Beckman Coulter
Vβ22	PE+FITC	IMMU546	Beckman Coulter
Vβ23	PE	AF23	Beckman Coulter
CD28	APC	CD28.2	Beckman Coulter
CD31	Pacific blue	5.6E	Beckman Coulter
CD4	APC-AF700	13B8.2	Beckman Coulter
CD45RA	APC-AF750	2H4	Beckman Coulter
CD8	Krome orange	B9.11	Beckman Coulter
TCRγδ	PC5.5	IMMU510	Beckman Coulter
CCR7	PE-Cy7	3D12	BD Biosciences

Overview of fluorochrome-conjugated monoclonal antibodies applied in the study.

### Calculation of Gini-TCR skewing index

Gini-TCR skewing index values were calculated for T cell subsets of individual blood donors with use of the Gini index, which is generally used to measure income distribution [7,8]. A Microsoft Excel file allowing automatic calculation of Gini-TCR skewing index from percentages of 24 TCR-Vβ families is provided in the Supporting Information (S1 File). A simplified overview to calculate the Gini-TCR skewing index is provided (S2 Fig). Briefly, proportions of all 24 TCR-Vβ families within a T cell subset were arranged from small to large. To construct Lorentz curves, the cumulative proportions of all TCR-Vβ family proportions were normalized to a total of 100%. Lorenz curves for individual blood donors were plotted with the cumulative percentage of the 24 TCR-Vβ families studied on the x-axis, and the cumulative proportion of CD4 or CD8 T cells that were covered by these 24 TCR-Vβ families on the y-axis. Next, the surface under the Lorenz curve was subtracted from the surface under the equidistribution line (i.e. line of perfectly equal distribution). To calculate the Gini-TCR skewing index, the surface between the equidistribution line and the Lorenz curve was divided by the total surface under the equidistribution line (100 * 100 / 0.5 = 5000) and then multiplied by 100. An example of the complete calculation of a Gini-TCR skewing index is shown in Table 2.

**Table 2:** Example showing calculation of Gini-TCR skewing index from TCR-Vβ usage of an individual blood donor.

#	Vβ usage (%)		Ranked Vβ usage (%)		Ranked Vβ usage (%)—Corrected to 100%	Cumulative TCR Vβ usage (%)	Surface under Lorenz curve	
1	*Vβ1*	3.23	*Vβ23*	0.58	0.78	0.78	*A*	3.25
2	*Vβ2*	10.84	*Vβ11*	0.77	1.03	1.81	*B*	5.40
3	*Vβ3*	7.62	*Vβ7*.*2*	0.78	1.05	2.86	*C*	9.74
4	*Vβ4*	2.68	*Vβ5*.*2*	0.92	1.24	4.10	*D*	14.49
5	*Vβ5*.*1*	5.65	*Vβ5*.*3*	0.92	1.24	5.33	*E*	19.64
6	*Vβ5*.*2*	0.92	*Vβ16*	1.18	1.58	6.92	*F*	25.52
7	*Vβ5*.*3*	0.92	*Vβ20*	1.48	1.99	8.90	*G*	32.96
8	*Vβ7*.*1*	1.97	*Vβ13*.*6*	1.56	2.10	11.00	*H*	41.47
9	*Vβ7*.*2*	0.78	*Vβ21*.*3*	1.88	2.52	13.52	*I*	51.09
10	*Vβ8*	3.84	*Vβ7*.*1*	1.97	2.65	16.17	*J*	61.86
11	*Vβ9*	4.72	*Vβ13*.*2*	2.13	2.86	19.03	*K*	73.33
12	*Vβ11*	0.77	*Vβ12*	2.22	2.98	22.01	*L*	85.50
13	*Vβ12*	2.22	*Vβ4*	2.68	3.60	25.61	*M*	99.21
14	*Vβ13*.*1*	3.34	*Vβ1*	3.23	4.34	29.95	*N*	115.75
15	*Vβ13*.*2*	2.13	*Vβ18*	3.33	4.47	34.42	*O*	134.10
16	*Vβ13*.*6*	1.56	*Vβ13*.*1*	3.34	4.49	38.91	*P*	152.77
17	*Vβ14*	3.41	*Vβ14*	3.41	4.58	43.49	*Q*	171.65
18	*Vβ16*	1.18	*Vβ22*	3.69	4.96	48.44	*R*	191.52
19	*Vβ17*	5.72	*Vβ8*	3.84	5.16	53.60	*S*	212.59
20	*Vβ18*	3.33	*Vβ9*	4.72	6.34	59.94	*T*	236.54
21	*Vβ20*	1.48	*Vβ5*.*1*	5.65	7.59	67.53	*U*	265.55
22	*Vβ21*.*3*	1.88	*Vβ17*	5.72	7.68	75.21	*V*	297.36
23	*Vβ22*	3.69	*Vβ3*	7.62	10.23	85.44	*W*	334.69
24	*Vβ23*	0.58	*Vβ2*	10.84	14.56	100.00	*X*	386.34
**Total**		**74.46**		**74.46**	**100.00**			**3022.32**

First TCR-Vβ families are ranked based on their proportional size. Then values are normalized to 100%. Subsequently, the surface under the Lorentz curve is calculated. In this blood donor, the surface between Lorenz curve and equidistribution line = 5000–3022.32 = 1977.68. Gini-TCR skewing index = 1977.68/5000*100 = 39.55.

### Hierarchical clustering analysis

Gini-TCR skewing index values of all T cell subsets from individual donors were plotted in a heat map using Microsoft Excel 2007. Colors were appointed based on absolute Gini-TCR skewing index values. Subsequently, hierarchical clustering analysis of T cell subsets and blood donors was performed in Genesis 1.7.6 (Genomics and Bioinformatics Graz) using Euclidean distance and compete linkage clustering.

### CMV ELISA

CMV-specific IgG was determined in serum samples using an in-house ELISA. 96-well ELISA plates (Greiner) were coated with lysates of CMV-infected fibroblasts overnight. Lysates of non-infected fibroblasts were used as negative controls. Following the coating, dilutions of serum samples were incubated for 1 hour. Goat anti-human IgG was added and incubated for 1 hour. Samples were incubated with phosphatase for 15 minutes and the reaction was stopped with NaOH. The plates were scanned on a Versamax reader (Molecular Devices). A pool of sera from 3 CMV-seropositive individuals with known concentrations of CMV-specific IgG was used to quantify levels of CMV-specific IgG in the tested samples.

### Vaccination

Nine year old children in good health were vaccinated with a combination vaccine against diphtheria, tetanus, acellular pertussis and inactivated poliovirus (DTaP-IPV). Peripheral blood mononuclear cells were isolated by density centrifugation before vaccination, as well as 28 days and 1 year after vaccination. Samples were viably stored at -135°C with RPMI + 10% FCS and 10% DMSO. To perform flow cytometric staining, the samples were thawed in a 37°C water bath and washed twice with RPMI + 10% FCS.

### Statistics

Gini-TCR skewing index values between two T cell subsets within individuals were compared with the Wilcoxon signed rank test. In case multiple T cell subsets were compared, the Wilcoxon signed rank test was preceded by the Friedman test. Gini-TCR skewing index values in CMV seropositive and seronegative individuals were compared with the Mann Whitney U test. Two-sided *p* values < 0.05 were considered statistically significant. Statistical analysis was performed with IBM SPSS Statistics 20 (SPSS, Chicago, IL, USA).

## Results

### Calculation of the Gini-TCR skewing index

**Fig 2:**
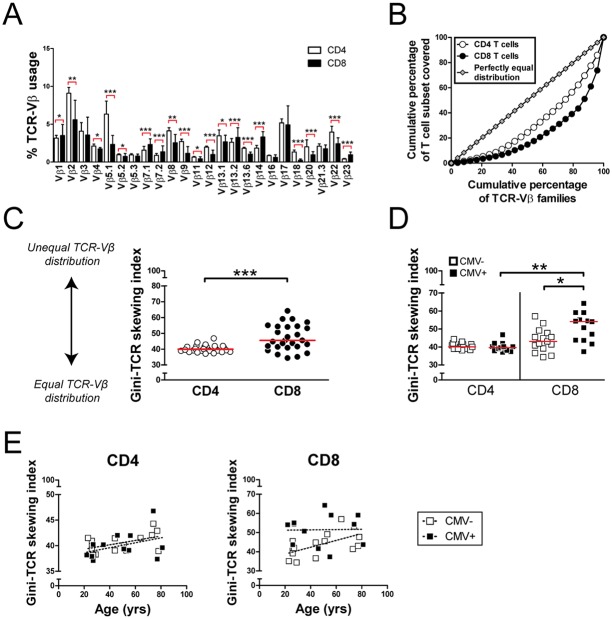
T cell receptor (TCR) Vβ distribution analysis in CD4 and CD8 T cells. (A) Traditional graph indicating proportional TCR-Vβ usage in CD4 and CD8 T cells of 27 healthy individuals. Bars and error bars represent median and interquartile ranges. (B) Lorenz curve of TCR-Vβ distribution in CD4 and CD8 T cells of a representative healthy individual. (C) Gini-TCR skewing index values in CD4 and CD8 T cells of 27 healthy individuals. Lines represent medians. (D) Gini-TCR skewing index values in CD4 and CD8 T cells of CMV-seropositive (CMV+) and seronegative (CMV-) individuals. Lines represent medians. (E) Relation between age and Gini-TCR skewing index values in CD4 and CD8 T cells from 27 healthy CMV+ and CMV- individuals. Statistical significance was tested with the Wilcoxon signed rank test or Mann Whitney U test and is indicated as * p<0.05, ** p<0.01, *** p<0.001.

To demonstrate that flow cytometry and economic statistics can be combined, we started by calculating Gini-TCR skewing index values for CD4 and CD8 T cells from healthy individuals. Therefore, we first measured the proportional usage of 24 TCR-Vβ families in CD4 and CD8 T cells with a commercially available panel of monoclonal antibodies (S1 Fig). In accordance with previous reports, the antibody panel recognized TCR-Vβ families on 66 ± 1% (mean ± SEM) of CD4 T cells and 60 ± 2% of CD8 T cells [12,13]. At this stage, it was difficult to conclude whether the distribution of TCR-Vβ families differed between CD4 and CD8 T cells (Fig 2A). Next, the proportional TCR-Vβ usage of CD4 and CD8 T cells from individual blood donors was plotted in Lorenz curves with the cumulative percentage of the 24 TCR-Vβ families studied on the x-axis, and the cumulative proportion of CD4 or CD8 T cells that was covered by these 24 TCR-Vβ families on the y-axis (Fig 2B and S2 Fig). A Lorenz curve far from the equidistribution line refers to unequal distribution and skewing of the TCR repertoire. Finally, Gini-TCR skewing index values were calculated for CD4 and CD8 T cells of individual blood donors (S2 Fig). In some individuals, Gini-TCR skewing index values were markedly higher for CD8 T cells than CD4 T cells, whereas the values were similar in others (Fig 2C). This difference could be explained by cytomegalovirus (CMV) infection, as only CD8 T cells of CMV-seropositive individuals demonstrated increased Gini-TCR skewing index values (Fig 2D). Furthermore, we noted that variation of Gini-TCR skewing index values in CMV-seronegative could be explained by age, as we observed a substantial, aging-associated increase in Gini-TCR skewing index values among CD8 T cells of CMV-seronegative individuals (Fig 2E). In contrast, the TCR-Vβ repertoire of CD8 T cells was already skewed in young CMV-seropositive donors and did not modulate further with age. In addition, Gini-TCR skewing index values only modestly increased with age among CD4 T cells, irrespective of CMV-serostatus. With these experiments, we demonstrated that flow cytometry can be combined with economic statistics and confirmed previous findings that CMV-infection and aging are associated with profound skewing of the TCR repertoire in CD8 T cells [14–16].

### Gini-TCR skewing index in T cell differentiation subsets

**Fig 3:**
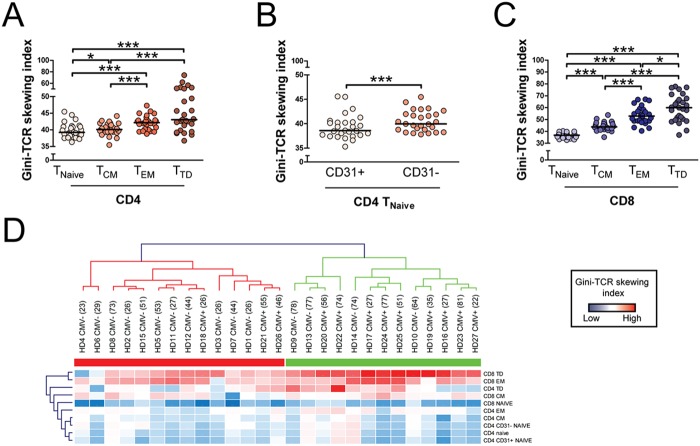
T cell receptor (TCR) Vβ distribution in CD4 and CD8 T cell subsets. (A) Gini-TCR skewing index values in naive (T_Naive_; CD45RA+CCR7+), central memory (T_CM_; CD45RA-CCR7+), effector memory (T_EM_; CD45RA-CCR7-) and terminally differentiated (T_TD_; CD45RA+CCR7-) CD4 T cells of 27 healthy individuals. (B) Gini-TCR skewing index values in CD31+ and CD31- naive CD4 T cells of 27 healthy individuals. (C) Gini-TCR skewing index values in CD8 T_Naive_, T_CM_, T_EM_ and T_TD_ cells of 27 healthy individuals. Lines represent medians. Statistical significance was tested with the Wilcoxon signed rank test and is indicated as * p<0.05, ** p<0.01, *** p<0.001. In case of multiple testing, the Wilcoxon signed rank test was preceded by the Friedman test. (D) Heat map based on absolute Gini-TCR skewing index values in CD4 and CD8 T cell differentiation subsets of healthy donors in which CMV status was determined. Age is shown between ‘()’. The dendrogram was based on hierarchical clustering analysis with use of Euclidean distance and complete linkage clustering.

To further validate our method, we determined Gini-TCR skewing index values in well-defined T cell differentiation subsets. T cells were therefore divided into naive (T_Naive_), central memory (T_CM_), effector memory (T_EM_) and terminally differentiated (T_TD_) T cells based on CD45RA and CCR7 expression [17] as shown in S3 Fig TCR sequencing and spectratyping studies have shown that naive T cells demonstrate the broadest TCR repertoire, whereas the repertoire becomes more contracted in memory T cells [18–20]. We indeed observed that CD4 T_Naive_ cells show significantly lower Gini-TCR skewing index values than the three CD4 memory cells subsets (Fig 3A). Among the memory cells, CD4 T_CM_ cells demonstrated significantly less TCR skewing than CD4 T_EM_ and T_TD_ cells, as previously found with TCR sequencing [18]. Further delineation of CD4 T_Naive_ cells based on CD31 expression (S3 Fig) confirmed the previous observation that CD31 negative T cells (central naive) have a slightly more skewed TCR repertoire than CD31 positive T cells (thymic naive) [21], as demonstrated in Fig 3B. Analysis of TCR-Vβ distribution in CD8 differentiation subsets (S3 Fig) resulted in similar findings as observed in CD4 T cells, although the TCR repertoire skewing was more pronounced in the CD8 T_EM_ and T_TD_ populations (Fig 3C). Taken together, calculation of the Gini-TCR skewing index in naive and memory T cell subsets provided similar results as previously reported with TCR sequencing and spectratyping [18–21].

### Hierarchical clustering analysis of Gini-TCR skewing index values

As the distribution of the TCR repertoire was simultaneously measured in a large number of distinct CD4 and CD8 T cell subsets, we next determined to what extent TCR skewing in one subset is linked to TCR skewing in another subset. Hierarchical clustering analysis readily revealed that CD8 T_EM_ and CD8 T_TD_ cells formed a separate cluster from all other CD4 and CD8 T cell subsets (Fig 3D). This finding indicates that TCR skewing in CD8 T_EM_ and T_TD_ cells occurs relatively independently from TCR skewing in the other subsets. An additional hierarchical clustering analysis showed that Gini-TCR skewing index values of CMV-seropositive and CMV-seronegative individuals largely formed two separate clusters. This finding further underscores the impact that CMV has on the TCR repertoire in humans [14].

### Tracking Gini-TCR skewing index changes following an antigenic challenge

**Fig 4:**
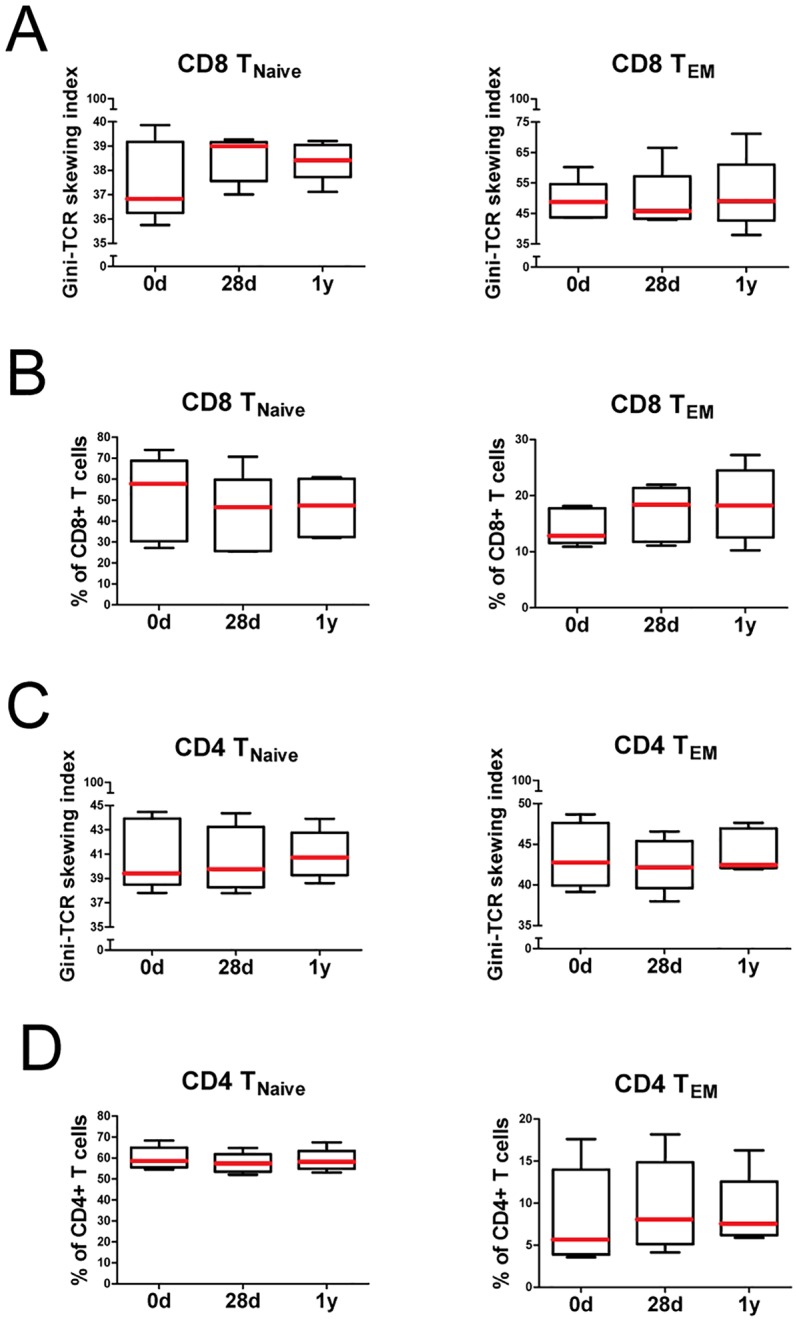
Analysis of naive and effector memory CD4 and CD8 T cells before and after DTaP-IPV vaccination. Five healthy children received a combination vaccine against diphtheria, tetanus, acellular pertussis and inactivated poliovirus (DTaP-IPV). Blood samples were collected before vaccination, and 28 days and 1 year after vaccination. (A) Gini-TCR skewing index values and (B) proportions of naive (T_Naive_) and effector memory (T_EM_) CD8 T cells before and after vaccination. (C) Gini-TCR skewing index values and (D) proportions of CD4 T_Naive_ and T_EM_ cells before and after vaccination. Boxes show median with interquartile range, whiskers are drawn according to the Tukey method.

Finally, we determined whether the Gini-TCR skewing index allowed us to detect TCR repertoire changes following an antigenic challenge. Therefore, we studied the TCR repertoire of CD4 and CD8 T_Naive_ and T_EM_ cells in 9-year old children receiving a combination vaccine against diphtheria, tetanus, acellular pertussis and inactivated poliovirus (DTaP-IPV). We hypothesized that DTaP-IPV vaccination will recruit naive T cells towards the effector memory T cell pool and thereby cause skewing of the naive TCR repertoire. In accordance with our expectations, we observed clear skewing of the TCR repertoire among CD8 T_Naive_ cells at 28 days after vaccination (Fig 4A, left panel). In parallel, we observed a slight decrease in Gini-TCR skewing index values, i.e. broadening of the TCR repertoire, among CD8 T_EM_ cells at 28 days (Fig 4A, right panel). The latter finding was only temporary, as Gini-TCR skewing index values of CD8 T_EM_ cells returned to baseline levels at 1 year after vaccination. In contrast, Gini-TCR skewing index values remained elevated among CD8 T_Naive_ cells after 1 year follow-up. These TCR repertoire changes were associated with a stable numerical decrease in CD8 T_Naive_ cells (Fig 4B, left panel) and increase in CD8 T_EM_ cells (Fig 4B, right panel). In essence, we observed comparable modulations within the CD4 T cell compartment (Fig 4C and 4D), albeit substantially less pronounced. These findings indeed implied that naive T cells are recruited towards the effector memory pool upon DTaP-IPV vaccination. Taken together, our results show that the Gini-TCR skewing index provides stable values over time, but is sensitive enough to detect TCR repertoire changes following an antigenic challenge.

## Discussion

We here report a novel application of the Gini index for analyzing flow cytometric data on the TCR-Vβ repertoire. Flow cytometry is widely used to investigate the human TCR repertoire, as it quickly provides insight into the TCR repertoire of many different T cell subsets without the need for laborious cell-sorting. Until now however, the analysis and interpretation of the flow cytometric data on TCR-Vβ usage was difficult and subtle TCR repertoire changes were easily missed [6]. In the current study, we show that economic statistics, which have previously been applied in TCR sequencing studies [10,11], allowed us to quantify the flow cytometric data on proportional TCR-Vβ usage into direct measures of TCR-Vβ distribution: the Gini-TCR skewing index. Using the Gini-TCR skewing index, we were capable of detecting subtle changes of the TCR repertoire among multiple, well-defined populations of T cells.

We validated the Gini-TCR skewing index by comparing our results to previous findings obtained with TCR sequencing and spectratyping [14,18–21]. We confirmed that chronic CMV infection and aging are associated with substantial skewing of the TCR repertoire in CD8 T cells [14–16]. Moreover, we found that naive and memory T cell populations display differences in TCR repertoire skewing. Naive T cells are known to have the broadest TCR repertoire, whereas the repertoire becomes skewed in memory T cells [18–20]. By calculating the Gini-TCR skewing index, we could confirm that naive T cells have a less skewed TCR-Vβ repertoire than memory T cells. In addition, we verified that CD31+ thymic naive CD4+ T cells have a broader TCR-Vβ repertoire than CD31- central naive CD4+ T cells [21]. Furthermore, we confirmed that central memory CD4 and CD8 T cells demonstrate less TCR repertoire skewing than effector memory and terminally differentiated memory T cells [18]. Interestingly, we could even detect changes of the TCR-Vβ repertoire following an antigenic challenge (i.e. DTaP-IPV vaccination). Our findings therefore confirm that distinct populations of T cells are characterized by distinct skewing of the TCR repertoire, and that these differences can be detected with the Gini-TCR skewing index. Furthermore, our data underscore that future TCR repertoire studies should take into account that age, antigenic challenges and the presence of chronic viral infections (i.e. CMV) have a profound impact on the TCR repertoire.

In summary, we introduce a novel and rapid approach for analyzing flow cytometric data on TCR-Vβ usage. By applying economic statistics, we could calculate the Gini-TCR skewing index and readily track subtle changes of the TCR-Vβ repertoire among a large number of T cell subsets. Our study therefore reveals interesting synergy between economic statistics and the flow cytometric analysis of the TCR repertoire. We expect that application of the Gini-TCR skewing index to the flow cytometric TCR-Vβ analysis will contribute to a better understanding of the TCR repertoire in health and disease. The development of novel monoclonal antibodies for additional TCR-Vβ families could make the flow cytometric assessment of the TCR repertoire also more suitable for use in clinical diagnostics [4,5,22].

## Supporting Information

S1 Fig Flow cytometric analysis of T cell receptor (TCR) Vβ usage. (A) Representative flow cytometric plot showing gating of CD4 T cells. Additional negative gating for CD8 T cells and γδ T cells was performed. (B) Representative flow cytometric plot showing gating of CD8 T cells. Additional negative gating for CD4 T cells and γδ T cells was performed. (C) Representative flow cytometric plots showing staining for 24 TCR-Vβ families in 8 separate tubes. Anti-TCR-Vβ antibodies were labeled with PE, FITC and PE+FITC.
(DOCX)

S2 Fig Simplified calculation of Gini-TCR skewing index based on the Gini index. A step-by-step explanation shows how to calculate Gini-TCR skewing index values. For clarity purposes, only 5 hypothetical TCR-Vβ families are shown instead of 24 TCR-Vβ families.
(DOCX)

S3 Fig Flow cytometric analysis of CD4 and CD8 T cell differentiation subsets. (A) Representative flow cytometric staining for CD45RA and CCR7 to identify naïve, central memory (CM), effector memory (EM) and terminally differentiated (TD) CD4 and CD8 T cells. The flow cytometric gates were based on fluorescence-minus-one (FMO) controls. (B) Representative flow cytometric staining for CD31 in CD4 T cells and FMO control for CD31.
(DOCX)

S1 File Microsoft Excel file allowing automatic calculation of the Gini-TCR skewing index. Percentages of the 24 TCR-Vβ families can be copied into the depicted section (colored cells). Following arrangement of values from low to high, the Gini-TCR skewing index will be calculated (in blue).
(XLSX)

## References

[1] Messaoudi I, Guevara Patino JA, Dyall R, LeMaoult J, Nikolich-Zugich J. Direct link between mhc polymorphism, T cell avidity, and diversity in immune defense. Science. 2002;298: 1797–1800. 12459592 10.1126/science.1076064

[2] Wang GC, Dash P, McCullers JA, Doherty PC, Thomas PG. T cell receptor alphabeta diversity inversely correlates with pathogen-specific antibody levels in human cytomegalovirus infection. Sci Transl Med. 2012;4: 128ra42 10.1126/scitranslmed.3003647 22491952 PMC3593633

[3] Saurwein-Teissl M, Lung TL, Marx F, Gschosser C, Asch E, Blasko I, et al Lack of antibody production following immunization in old age: Association with CD8(+)CD28(-) T cell clonal expansions and an imbalance in the production of Th1 and Th2 cytokines. J Immunol. 2002;168: 5893–5899. 12023394 10.4049/jimmunol.168.11.5893

[4] Gorochov G, Neumann AU, Kereveur A, Parizot C, Li T, Katlama C, et al Perturbation of CD4+ and CD8+ T-cell repertoires during progression to AIDS and regulation of the CD4+ repertoire during antiviral therapy. Nat Med. 1998;4: 215–221. 9461196 10.1038/nm0298-215

[5] Manuel M, Tredan O, Bachelot T, Clapisson G, Courtier A, Parmentier G, et al Lymphopenia combined with low TCR diversity (divpenia) predicts poor overall survival in metastatic breast cancer patients. Oncoimmunology. 2012;1: 432–440. 22754761 10.4161/onci.19545 PMC3382902

[6] Miles JJ, Douek DC, Price DA. Bias in the alphabeta T-cell repertoire: Implications for disease pathogenesis and vaccination. Immunol Cell Biol. 2011;89: 375–387. 10.1038/icb.2010.139 21301479

[7] Ceriani L, Verme P. The origins of the gini index: Extracts from variabilità e mutabilità (1912) by corrado gini. The Journal of Economical Inequality. 2012;10: 421–443.

[8] Bellù LG, Liberati P. Inequality analysis: The gini index. Available: http://www.fao.org/easypol/output/AbsLanguage.asp?id=329&job_no=040&langa=EN. 2005. Accessed March 2015.

[9] Gastwirth JL. The estimation of the lorenz curve and gini index. The Review of Economics and Statistics. 1972;54: 306.

[10] Laydon DJ, Melamed A, Sim A, Gillet NA, Sim K, Darko S, et al Quantification of HTLV-1 clonality and TCR diversity. PLoS Comput Biol. 2014;10: e1003646 10.1371/journal.pcbi.1003646 24945836 PMC4063693

[11] Thomas PG, Handel A, Doherty PC, La Gruta NL. Ecological analysis of antigen-specific CTL repertoires defines the relationship between naive and immune T-cell populations. Proc Natl Acad Sci U S A. 2013;110: 1839–1844. 10.1073/pnas.1222149110 23319654 PMC3562793

[12] Langerak AW, van Den Beemd R, Wolvers-Tettero IL, Boor PP, van Lochem EG, Hooijkaas H, et al Molecular and flow cytometric analysis of the vbeta repertoire for clonality assessment in mature TCRalphabeta T-cell proliferations. Blood. 2001;98: 165–173. 11418476 10.1182/blood.v98.1.165

[13] De Rosa SC, Herzenberg LA, Herzenberg LA, Roederer M. 11-color, 13-parameter flow cytometry: Identification of human naive T cells by phenotype, function, and T-cell receptor diversity. Nat Med. 2001;7: 245–248. 11175858 10.1038/84701

[14] Khan N, Shariff N, Cobbold M, Bruton R, Ainsworth JA, Sinclair AJ, et al Cytomegalovirus seropositivity drives the CD8 T cell repertoire toward greater clonality in healthy elderly individuals. J Immunol. 2002;169: 1984–1992. 12165524 10.4049/jimmunol.169.4.1984

[15] Britanova OV, Putintseva EV, Shugay M, Merzlyak EM, Turchaninova MA, Staroverov DB, et al Age-related decrease in TCR repertoire diversity measured with deep and normalized sequence profiling. J Immunol. 2014;192: 2689–2698. 10.4049/jimmunol.1302064 24510963

[16] Qi Q, Liu Y, Cheng Y, Glanville J, Zhang D, Lee JY, et al Diversity and clonal selection in the human T-cell repertoire. Proc Natl Acad Sci U S A. 2014;111: 13139–13144. 10.1073/pnas.1409155111 25157137 PMC4246948

[17] Sallusto F, Lenig D, Forster R, Lipp M, Lanzavecchia A. Two subsets of memory T lymphocytes with distinct homing potentials and effector functions. Nature. 1999;401: 708–712. 10537110 10.1038/44385

[18] Baum PD, Young JJ, Schmidt D, Zhang Q, Hoh R, Busch M, et al Blood T-cell receptor diversity decreases during the course of HIV infection, but the potential for a diverse repertoire persists. Blood. 2012;119: 3469–3477. 10.1182/blood-2011-11-395384 22371879 PMC3325037

[19] Klarenbeek PL, Tak PP, van Schaik BD, Zwinderman AH, Jakobs ME, Zhang Z, et al Human T-cell memory consists mainly of unexpanded clones. Immunol Lett. 2010;133: 42–48. 10.1016/j.imlet.2010.06.011 20621124

[20] Robins HS, Campregher PV, Srivastava SK, Wacher A, Turtle CJ, Kahsai O, et al Comprehensive assessment of T-cell receptor beta-chain diversity in alphabeta T cells. Blood. 2009;114: 4099–4107. 10.1182/blood-2009-04-217604 19706884 PMC2774550

[21] Kohler S, Wagner U, Pierer M, Kimmig S, Oppmann B, Mowes B, et al Post-thymic in vivo proliferation of naive CD4+ T cells constrains the TCR repertoire in healthy human adults. Eur J Immunol. 2005;35: 1987–1994. 15909312 10.1002/eji.200526181

[22] van Heijst JW, Ceberio I, Lipuma LB, Samilo DW, Wasilewski GD, Gonzales AM, et al Quantitative assessment of T cell repertoire recovery after hematopoietic stem cell transplantation. Nat Med. 2013;19: 372–377. 10.1038/nm.3100 23435170 PMC3594333

