# Supplementary material for: Quantifying Distribution of Flow Cytometric TCR-Vβ Usage with Economic Statistics
Source: PLoS One. 2015 Apr 29;10(4):e0125373. doi: 10.1371/journal.pone.0125373 (PMC4414620; doi:10.1371/journal.pone.0125373)

**S1 Fig. Flow cytometric analysis of T cell receptor (TCR) Vβ usage.** (A) Representative flow cytometric plot showing gating of CD4 T cells. Additional negative gating for CD8 T cells and γδ T cells was performed. (B) Representative flow cytometric plot showing gating of CD8 T cells. Additional negative gating for CD4 T cells and γδ T cells was performed. (C) Representative flow cytometric plots showing staining for 24 TCR-Vβ families in 8 separate tubes. Anti-TCR-Vβ antibodies were labeled with PE, FITC and PE+FITC.


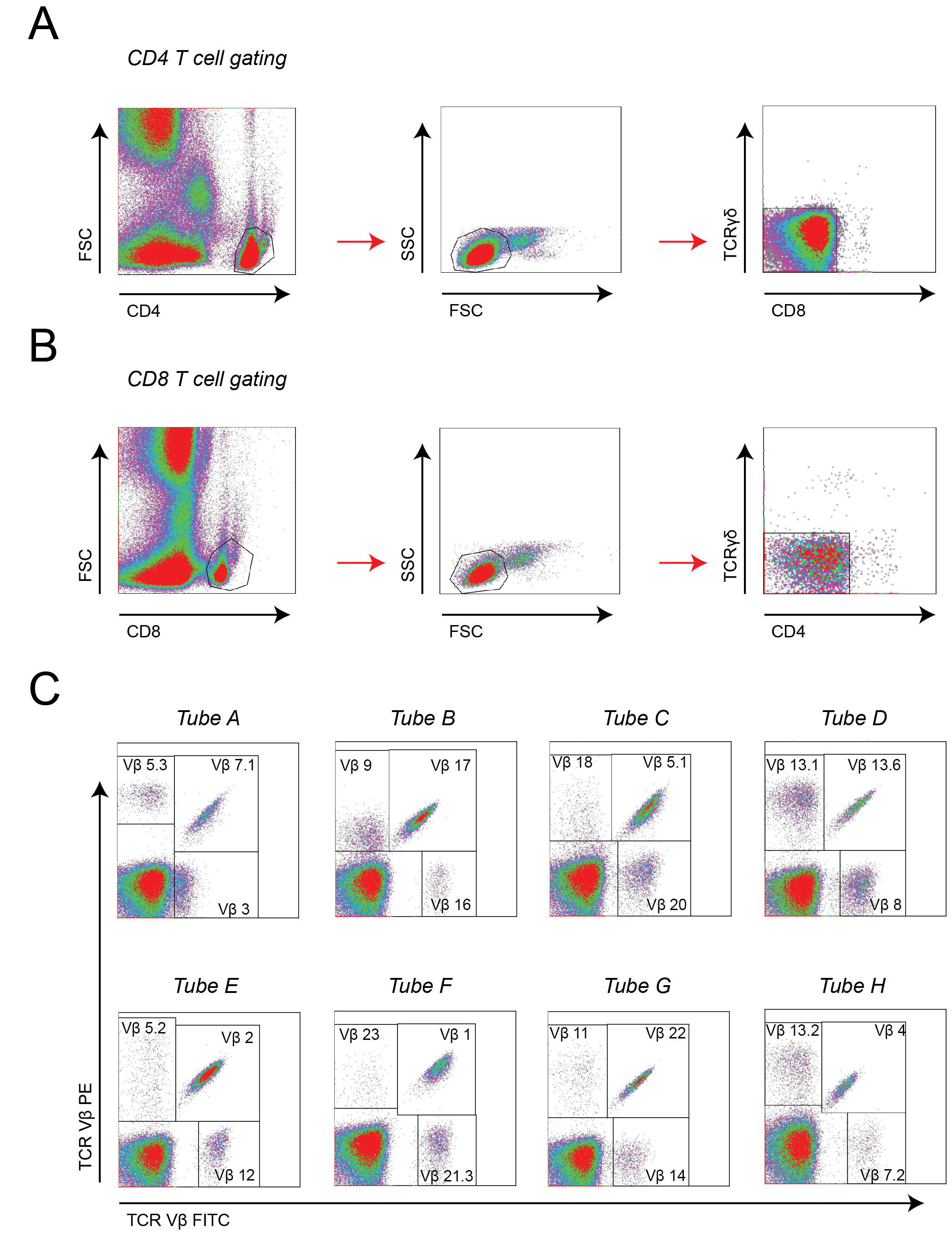

Supplement: S1 Fig — (A) Representative flow cytometric plot showing gating of CD4 T cells. Additional negative gating for CD8 T cells and γδ T cells was performed. (B) Representative flow cytometric plot showing gating of CD8 T cells. Additional negative gating for CD4 T cells and γδ T cells was performed. (C) Representative flow cytometric plots showing staining for 24 TCR-Vβ families in 8 separate tubes. Anti-TCR-Vβ antibodies were labeled with PE, FITC and PE+FITC. (DOCX) [file pone.0125373.s001.docx]
