# Supplementary material for: Quantifying Distribution of Flow Cytometric TCR-Vβ Usage with Economic Statistics
Source: PLoS One. 2015 Apr 29;10(4):e0125373. doi: 10.1371/journal.pone.0125373 (PMC4414620; doi:10.1371/journal.pone.0125373)

**S2 Fig. Simplified calculation of Gini-TCR skewing index based on the Gini index.** A step-by-step explanation shows how to calculate Gini-TCR skewing index values. For clarity purposes, only 5 hypothetical TCR-Vβ families are shown instead of 24 TCR-Vβ families.


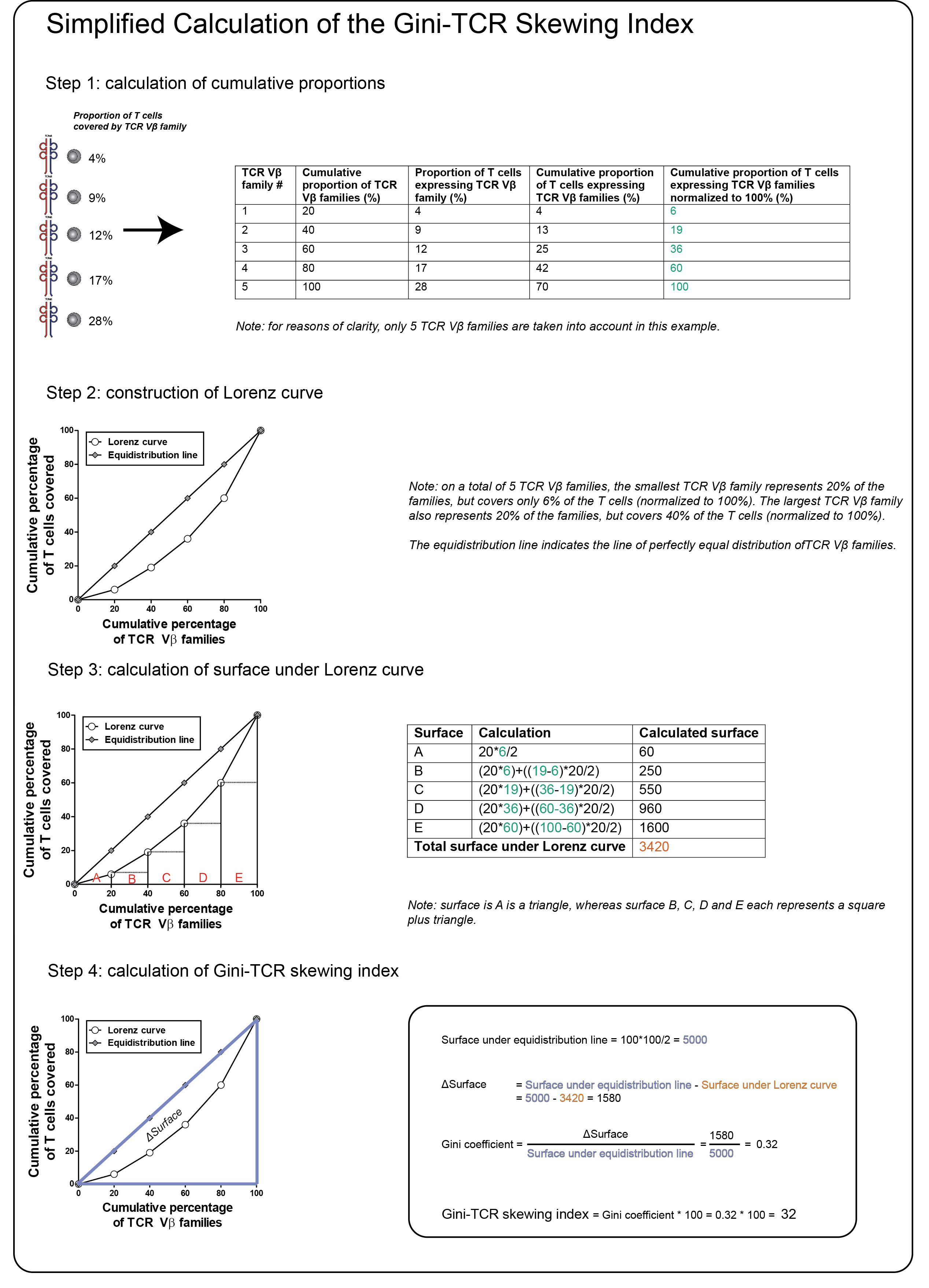

Supplement: S2 Fig — A step-by-step explanation shows how to calculate Gini-TCR skewing index values. For clarity purposes, only 5 hypothetical TCR-Vβ families are shown instead of 24 TCR-Vβ families. (DOCX) [file pone.0125373.s002.docx]
