# Supplementary material for: Quantifying Distribution of Flow Cytometric TCR-Vβ Usage with Economic Statistics
Source: PLoS One. 2015 Apr 29;10(4):e0125373. doi: 10.1371/journal.pone.0125373 (PMC4414620; doi:10.1371/journal.pone.0125373)

**S3 Fig. Flow cytometric analysis of CD4 and CD8 T cell differentiation subsets.** (A) Representative flow cytometric staining for CD45RA and CCR7 to identify naïve, central memory (CM), effector memory (EM) and terminally differentiated (TD) CD4 and CD8 T cells. The flow cytometric gates were based on fluorescence-minus-one (FMO) controls. (B) Representative flow cytometric staining for CD31 in CD4 T cells and FMO control for CD31.


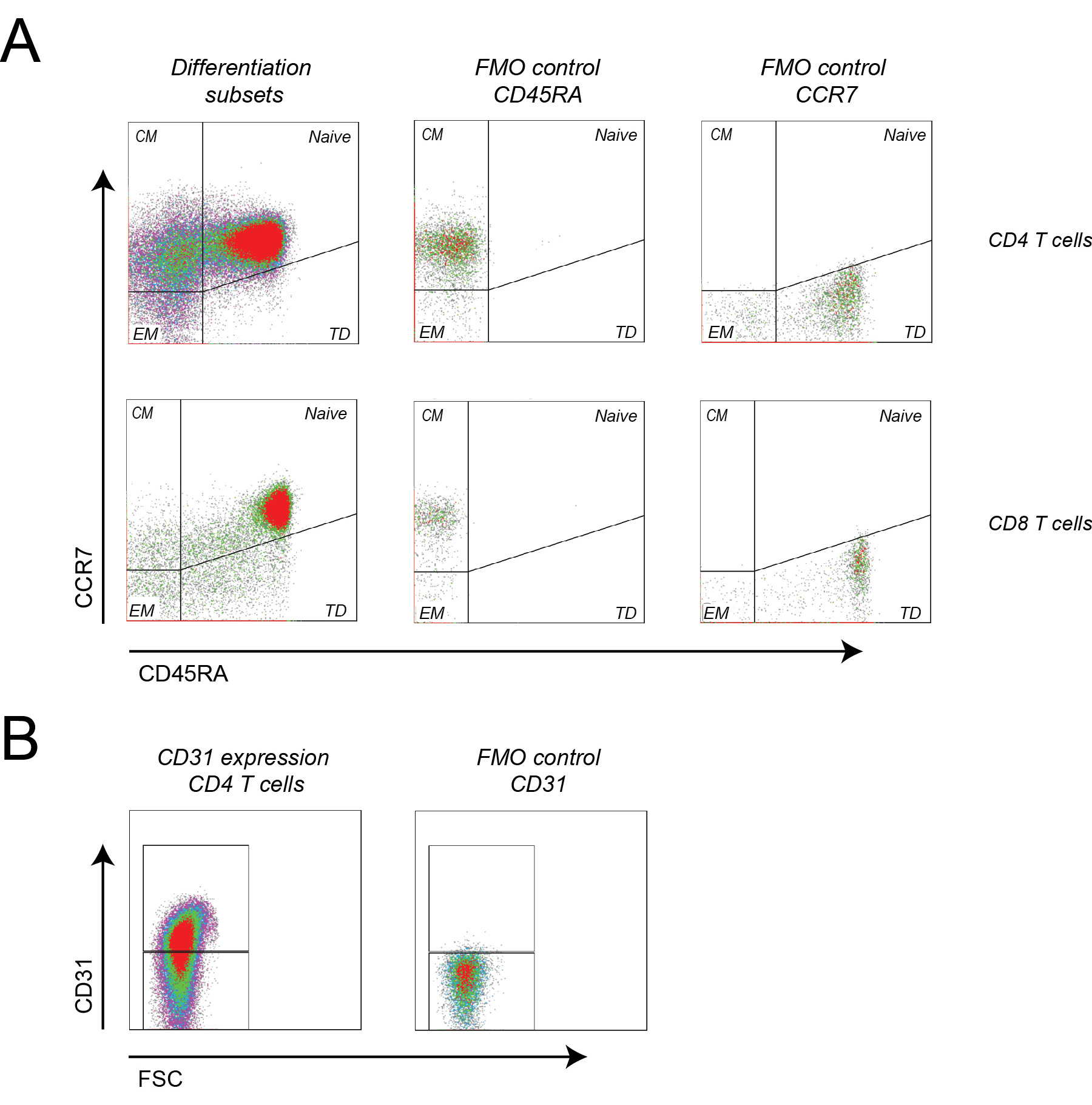

Supplement: S3 Fig — (A) Representative flow cytometric staining for CD45RA and CCR7 to identify naïve, central memory (CM), effector memory (EM) and terminally differentiated (TD) CD4 and CD8 T cells. The flow cytometric gates were based on fluorescence-minus-one (FMO) controls. (B) Representative flow cytometric staining for CD31 in CD4 T cells and FMO control for CD31. (DOCX) [file pone.0125373.s003.docx]
